# A2TEA: Identifying trait-specific evolutionary adaptations

**DOI:** 10.12688/f1000research.126463.1

**Published:** 2022-10-05

**Authors:** Tyll Stöcker, Carolin Uebermuth-Feldhaus, Florian Boecker, Heiko Schoof

**Affiliations:** 1 Crop Bioinformatics, University of Bonn, Bonn, NRW, 53115, Germany

**Keywords:** plants, crops, adaptation, evolution, stress, workflow, software

## Abstract

**Background:** Plants differ in their ability to cope with external stresses (e.g., drought tolerance). Genome duplications are an important mechanism to enable plant adaptation. This leads to characteristic footprints in the genome, such as protein family expansion. We explore genetic diversity and uncover evolutionary adaptation to stresses by exploiting genome comparisons between stress tolerant and sensitive species and RNA-Seq data sets from stress experiments. Expanded gene families that are stress-responsive based on differential expression analysis could hint at species or clade-specific adaptation, making these gene families exciting candidates for follow-up tolerance studies and crop improvement.

**Software:** Integration of such cross-species omics data is a challenging task, requiring various steps of transformation and filtering. Ultimately, visualization is crucial for quality control and interpretation. To address this, we developed A2TEA: Automated Assessment of Trait-specific Evolutionary Adaptations, a Snakemake workflow for detecting adaptation footprints in silico. It functions as a one-stop processing pipeline, integrating protein family, phylogeny, expression, and protein function analysis. The pipeline is accompanied by an R Shiny web application that

allows exploring, highlighting, and exporting the results interactively. This allows the user to formulate hypotheses regarding the genomic adaptations of one or a subset of the investigated species to a given stress.

**Conclusions: **While our research focus is on crops, the pipeline is entirely independent of the underlying species and can be used with any set of species. We demonstrate pipeline efficiency on real-world datasets and discuss the implementation and limits of our analysis workflow as well as planned extensions to its current state. The A2TEA workflow and web application are publicly available at: https://github.com/tgstoecker/A2TEA.Workflow and https://github.com/tgstoecker/A2TEA.WebApp, respectively.

## Introduction

**Figure 1.**
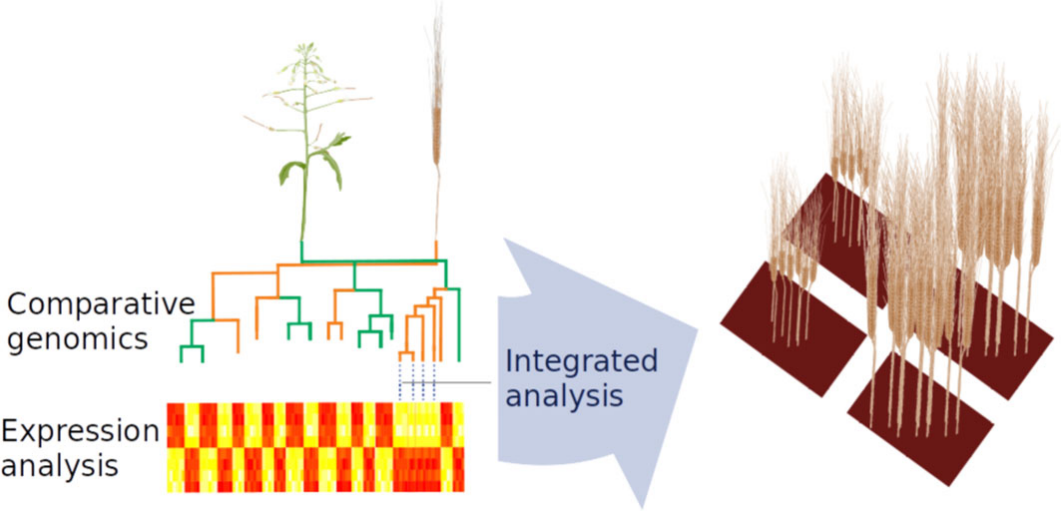
Identification of interesting gene families for crop improvement by integration of differential gene expression with gene family expansion.

While genomic resources are being expanded in crop species, with more and more high-quality reference genome sequences and transcriptome datasets becoming available, the lack of integrated trait and functional information limits the ability to interpret genomic-scale datasets and discover genotype-phenotype associations. While many efforts have focussed on individual (model) species, the availability of omics data for many more or less related genomes opens opportunities to explore genetic diversity through multi-genome comparisons. While differential expression analysis has led to the discovery of many candidate genes involved in tolerance to stresses, we aim to prioritize genes that were targets of evolutionary adaptation, thus facilitating application in crop improvement. To identify genomic footprints of adaptation, we use comparative genomics to identify protein family expansions. Gene duplication is a major driver of molecular evolution, ^1^^,^^2^ and in plants, whole-genome duplication events are frequent (reviewed in Ref. 3), but tandem and transposon-mediated duplications also play a role (reviewed in Ref. 4). Most of the duplicates are lost or silenced, ^5^ but which duplicates are retained hints at some evolutionary advantage. These may be targets of adaptation. ^6^ Especially for the adaptation of regulatory networks, duplication allows for neo- or subfunctionalization. ^7^ These form an evolutionary scenario that can be observed based on our integration of phylogeny and differential expression under stress data. However, we also consider differential expression under a given stress in any species as a functional link to the given stress, even if there is not sufficient data to confirm neo- or subfunctionalization. This allows us to filter for gene family expansions functionally linked to the given stress, as not all adaptations and thus retained duplicates in a genome need to relate to tolerance to the given stress, other traits not under analysis will also show adaptation and thus protein family expansions ( Figure 1).

The challenge for this multi-genome approach is the cross-species integration of multiple types of omics data, which requires several software tools and various custom steps of transformation and filtering. To promote the exploration of genomics and transcriptomics data and the association of genotype with phenotype data in order to address adaptation, we developed **A2TEA** ( **A**utomated **A**ssessment of **T**rait-specific **E**volutionary **A**daptations). Our software aims at identifying candidate genes for stress adaptation in plant species and enables GUI-based exploration of the results, but is suitable for gene family expansion analysis integrated with differential expression data in any set of genomes. It is composed of a Snakemake workflow and an R (Shiny) package working in tandem to automate and ease all bioinformatics & analysis tasks involved.

The A2TEA.Workflow functions as a one-stop processing pipeline, integrating the prediction of gene families in form of orthologous groups (OGs) with the analysis of their phylogeny, protein function, and expression, using RNA-seq data from all species. It allows the user to formulate adaptation hypotheses as specific scenarios of gene family expansion in one or several of the genomes, for example, based on a classification of species as stress-tolerant or sensitive, or to identify clade-specific adaptations. As input, the workflow requires for each species a protein FASTA file for orthologous group prediction and RNA-seq reads suitable for differential expression analysis (control vs. treatment), together with either a genomic FASTA file with appropriate gene annotation or a transcriptomic/cDNA FASTA file. Functional information for each species can be provided by the user or can be optionally inferred by our tool AHRD ( https://github.com/groupschoof/AHRD) during runtime. The single compressed output is ready for analysis with the R programming language ^8^ as we took care to create well-structured objects and easy-to-parse outputs. In addition, in order to facilitate immediate and easy exploration and visualization of the results, we created the A2TEA.WebApp written in R Shiny, ^9^ which allows exploring, highlighting, and exporting the results interactively.

The A2TEA.Workflow combines state-of-the-art bioinformatics software with custom integration steps to combine inferred gene family expansion events with expression results and functional associations.

The workflow is designed as a complete solution starting with raw data and performing upstream quality controls and data transformations automatically. We also took care to allow for a high degree of customizability - e.g. RNA-seq analysis can be performed either alignment-based or using pseudoalignment, and tool-specific parameters can be tweaked in one central config file. Importantly, the workflow is designed to answer biological questions and as such requires the definition of hypotheses in form of combinations of the species of interest. For each hypothesis, the user needs to adjust parameters related to the definition and cutoffs of expansion events. This allows computation of results for several combinations in parallel and facilitates the investigation of many hypotheses downstream e.g., expansion in all tolerant species, in only a specific species, or in all species of a clade.

The A2TEA.WebApp provides an interactive web interface to explore, filter, and visualize the previously generated results via a straightforward tab-structured dashboard design. We took care to create a user-friendly mouse-controlled experience in order to extend the usability from bioinformaticians to experimentalists. The user first uploads the output file of the workflow and chooses the specific “hypothesis” to investigate. This generates a general information tab providing an overview of phylogeny, expression, and set sizes of orthologous groups (OGs) passing the thresholds. The user is then able to switch to dedicated analysis tabs relating to 1) filtering and analyzing OGs with associated data, 2) set size comparisons and tests, and 3) gene ontology (GO) term enrichment analyses. Reactively rendered tables and visualizations are dynamically populated with links to databases such as Ensembl ^10^ and AmiGO ^11^ to allow for an immediate follow-up exploration of interesting genes. Tables and graphs can be exported in a variety of formats. The web application also provides a bookmarking system that facilitates the collection and export of the most interesting genes and OGs.

To extend the usability of the workflow by allowing for further species-specific exploration of gene and geneset functional enrichments we integrated the creation of GeneTonic input data files into the A2TEA.Workflow. GeneTonic is a web application that serves as a comprehensive toolkit for streamlining the interpretation of functional enrichment analyses from RNA-seq data. ^12^ As our workflow is built on Snakemake, ^13^ the addition of further analyses or outputs allows for modular expansion of its current state. We also intend to add further analyses and features to the A2TEA.WebApp web application.

A2TEA combines best practices in both choice of tools as well as reproducibility and offers a one-stop solution for the integration of genome comparisons with expression and functional data to unravel candidate genes for natural adaptation, e.g. in stress-tolerant plant species. The web application empowers users to explore stress-specific gene family expansions combined with transcriptomic data from their own or published stress experiments by providing interactive visualizations, statistical tests, and dynamically generated database queries.

Both the A2TEA.Workflow and A2TEA.WebApp are freely available at https://github.com/tgstoecker/A2TEA.Workflow and https://github.com/tgstoecker/A2TEA.WebApp, respectively, and archived in Zenodo. ^51^^,^^52^ For demonstration purposes, we also made a public instance of the web application available at https://tgstoecker.shinyapps.io/A2TEA-WebApp.

## Methods

### Implementation

The A2TEA.Workflow is written in Python and makes use of the Snakemake workflow framework. It leverages the bioconda project channel ^14^ of the conda package manager to handle software installation and dependency management. Another tool from our lab, AHRD, is integrated as a Git submodule and can be optionally used to infer protein function annotation for any of the species under investigation.

**Figure 2.**
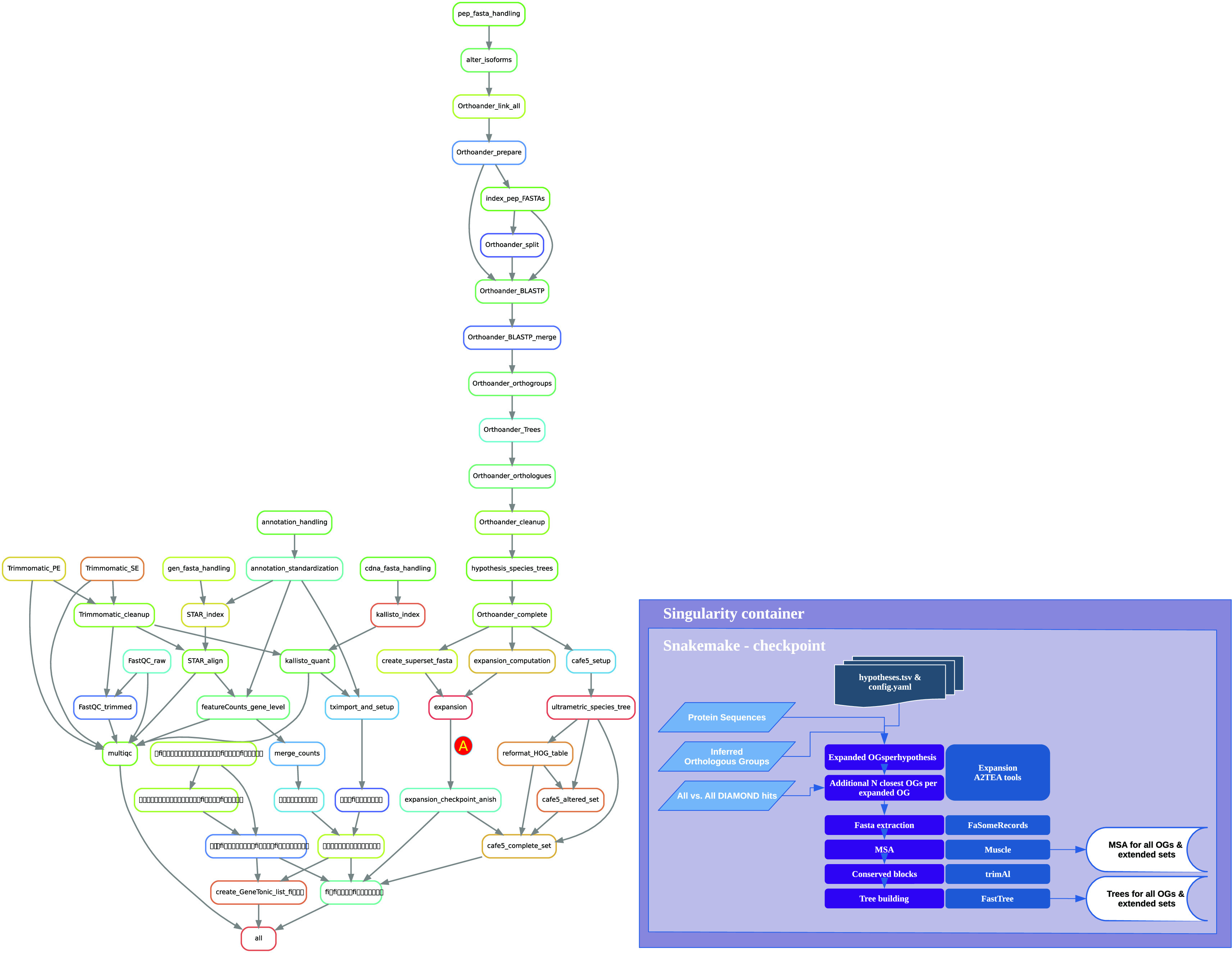
Overview of the A2TEA.Workflow. Workflow diagram of the A2TEA.Workflow displayed as Snakemake rulegraph. After computation of expanded orthologous groups (OGs) (rule expansion - marked with A) the directed acyclic graph (DAG) is re-evaluated since the results are not known beforehand. This Snakemake checkpoint then uses the reciprocal best hits computed by Orthofinder to find the N most similar additional OGs per OG, where N is a variable set by the user. For each OG and additional set of 1 to N additional OGs, multiple sequence alignments and phylogenetic trees are built and used in the downstream steps.

The typical use case for running the workflow consists of cloning the GitHub repository, configuring it to specific needs, and then starting the analyses with either installation of software and dependencies during runtime or usage of a Docker/Singularity container ( Figure 2). Modification of the workflow is performed by changing dedicated configuration files controlling samples, species, hypotheses, and tool-specific options. With “hypotheses” we refer to the definition of “gene family expansion” in the set of species under investigation. Several hypotheses can be run in parallel. This multi-hypothesis structure permits the investigation of several defined biological questions, for instance, gene family expansion in stress-tolerant compared to stress-sensitive species. For each hypothesis, we always require the definition of a set of one or more species that should be checked for expansion compared to a second set of one or more species that should not show expansion. For each hypothesis, the user is able to set several options, such as the ratio or the minimal number of genes in a species, to qualify as an expanded OG. The hypotheses.tsv file is structured column-wise with both an index number and a “name” variable used to identify the choices throughout the workflow. Generally, the connection between files and workflow rules is achieved by the species names (e.g., “Arabidopsis_thaliana”). Many hypotheses can be computed in a single workflow with a single final output object that contains all results. This facilitates easy comparisons in the downstream web application, which is especially useful to check the parameter choices for the definition of gene family expansion or when it is necessary to work with unclear trait classification of some species.

The final output generated by the workflow is a single .RData file that can be loaded into an active R environment with the load() command. This provides several separate objects containing all results in a compact form factor:

• HYPOTHESES.a2tea - List object with one S4 object per hypothesis. Each S4 object contains several layers of nested information. E.g. HYPOTHESES.a2tea$hypothesis_2@expanded_OGs$N0.HOG0001225 refers to a specific expanded OG and S4 data object that contains:
  – blast_table (complete BLAST/DIAMOND results for OG genes & extended hits)
  – add_OG_analysis (includes multiple sequence alignment (MSA), phylogenetic tree, and gene info for expanded OG and additional OGs based on best BLAST/DIAMOND hits)
• HOG_level_list - List object with one tibble per hypothesis. Information includes OG, number of genes per species, boolean expansion info, number of significant differentially expressed genes (DEGs), and more. The last N list element is a non-redundant superset of all species analyzed over all formulated hypotheses. This makes it easy to create a comparison set e.g., conserved OGs of all species to which the hypothesis subset can then be compared.
• HOG_DE.a2tea - Tibble of DESeq2 results for all genes + additional columns.
• A2TEA.fa.seqs - Non-redundant list object containing corresponding amino acid FASTA sequences of all genes/transcripts in the final analysis (this includes those of expanded OGs + those in additional BLAST hits & additional OGs based on user-chosen parameters).
• SFA/SFA_OG_level - Gene/transcript level tables that contain functional predictions (human readable descriptions & GO terms inferred by AHRD).
• hypotheses - A copy of the user-defined hypotheses definitions for the underlying workflow run.
• all_speciesTree - Phylogenetic tree of all species in the workflow run (a non-redundant superset of hypotheses) as inferred by Orthofinder/STAG/Stride.

The .RData output can be investigated inside an R session or via the A2TEA.WebApp, which was specifically designed to allow for interactive inspection, visualization, filtering, and export of the results and subsets. We feature a tutorial for its usage and details on how to work with the results of an A2TEA.Workflow analysis run in the Use Case section and in the project’s pkgdown site.

The A2TEA.WebApp is written in the R programming language ^8^ and uses the Shiny ^9^ framework to facilitate interactivity with the data. It expects the user to upload an .RData file created by the A2TEA.Workflow. The web application comes with a test dataset that can be loaded with a single click so that interested users can try out its functionality before having to finish an A2TEA.Workflow run.

We developed the web application following community standards and have set up a continuous integration system with GitHub actions that performs build checks of both the package itself and the associated pkgdown site ^15^ hosted via GitHub pages.

The A2TEA.WebApp is written in the R programming language and uses the Shiny framework to facilitate interactivity with the data. It expects the user to upload a .RData file created by the A2TEA.Workflow. The web application comes with a test dataset that can be loaded with a single click so interested users can try out its functionality before having to finish an A2TEA.Workflow run. We developed the web application following community standards and have set up a continuous integration system with GitHub actions that performs build checks of both the package itself and the associated pkgdown site (Wickham et al., 2022) ^15^ hosted via GitHub pages.

**Figure 3.**
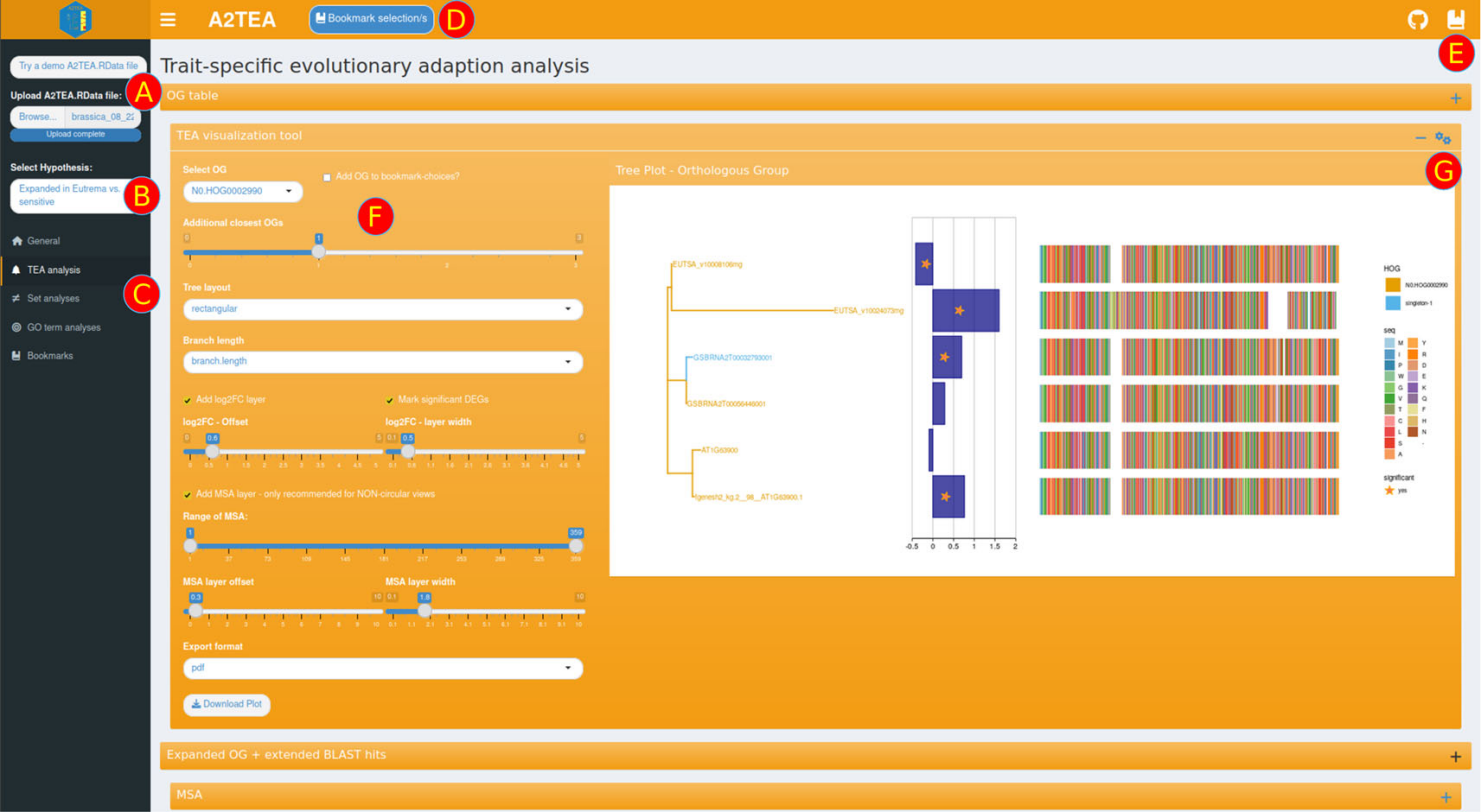
Screenshot of the trait evolutionary analysis tab in the A2TEA.WebApp. The user can either decide to load the included test dataset or upload a .RData result object (A). Other options in the sidebar menu are a selector of the hypothesis (meaning: species comparison) to display or change to another analysis tab (C). Genes, transcripts, or orthologous groups (OGs) can be marked in tables or boxes ticked and then bookmarked with a dedicated button (D). Bookmarks have their dedicated tab but can also be displayed as a sidebar window anywhere for quick reference purposes (E). Analysis- or plot-specific parameters (F) are displayed to the left of the visualization, and a box-specific sidebar window for aesthetic parameters can be opened by clicking the gears icon (G). The underlying dataset investigates drought tolerance among four Brassicaceae species. Displayed here are the maximum likelihood phylogenies of a gene family showing potential subfunctionalization of Eutrema salsugineum homologs (top two genes in the tree). Blue bars show log2 (fold change) of gene expression between drought and control conditions, stars mark adj. p ≤ 0.1 significance cutoff, and the multiple sequence alignment of amino acid sequences is displayed to the right. We provide this particular OG as a bookmarked subset .RData file (see Underlying Data).

The interface is structured in tabs with shinydashboard ^16^ and shinydashboardPlus, ^17^ providing the layout infrastructure (shown in Figure 3). The main functionality includes a selector to choose which hypothesis to display ( Figure 3B), a sidebar menu that enables the user to switch between different analysis types ( Figure 3C), and tool-specific options for parameters, visuals, and export ( Figure 3F).

We designed the interface to allow the focus to be put on an individual analysis or plot to gain insight from the data. Plots and tables are contained in collapsible boxes, leaving it up to the user to decide how much information should be displayed at once. Additionally, we tried to separate important parameters from purely aesthetic choices in the plot options, with main options always visible at the side and aesthetic choices reactively displayed after the user switches a box toggle.

Since the exploration of data can be a lengthy process with many iteration cycles, we looked for ways of aiding the user in storing the observations made. Following the example set by the GeneTonic web application, ^12^ we integrated a bookmarking system that temporarily stores interesting genes/transcripts and OGs. For this, the user needs to mark the respective ID in one of the tables and then click the dedicated bookmarking button displayed at the top of the interface ( Figure 3D). All bookmarks are rendered in two reference tables, both in a dedicated tab as well as a pop-up window that can be displayed on every analysis tab. This quick reference is convenient when performing filtering operations in the tables or choosing an interesting OG to display. While these tables can of course, be downloaded, we also implemented a subsetting feature on the bookmarks tab that creates a smaller .RData file with information only pertaining to the bookmarks and associated data. These smaller subsets are fully functioning complete inputs and can be loaded into the application again at a later time - for re-plotting purposes for instance. With this feature, it is straightforward to extract, store and share all information about interesting genes, transcripts, or OGs.

### Operation

The A2TEA.Workflow has been primarily designed for use within Linux and requires a standard bash environment with working installations of Snakemake ^13^ and conda/mamba (Ref. 14, https://github.com/mamba-org/mamba). The former facilitates compatibility with common cluster setups such as SLURM or LSF. Instructions for a minimal setup are described in the project’s README.

At the moment, for each species, the A2TEA.Workflow requires as input RNA-Seq reads (both paired-/single-end possible) suitable for differential expression analysis (control vs. treatment), either a genomic or transcriptomic FASTA file, annotations, and a peptide FASTA file. The user can provide functional information per species, or it can be optionally inferred by our tool AHRD during the workflow. Control of the workflow is handled by several configuration files, which the user needs to adapt to their specific inputs and scientific questions.

The samples.tsv table needs to list all RNA-seq FASTQ files with the columns providing additional information based on which the workflow can infer associations such as species, replicate, and the correct steps to perform. For instance, by leaving out the column for the second paired sample, it is automatically inferred that single-end options have to be used (trimming, mapping, etc.). Operations such as recognizing that files are gzipped and need to be handled appropriately are performed automatically as well. The species.tsv table functions similarly and needs to provide per species information on the FASTA and annotation files, the ploidy of the species, and the location of a file providing the functional information, in the form of GO terms, per protein. If no functional information can be provided the user can choose to add “AHRD” instead of a file path which will trigger a sub-workflow during computation that will create an appropriate file via our functional annotation tool AHRD. Based on whether the user provides a genomic or cDNA FASTA file for a species, the workflow will perform either traditional alignment with STAR ^18^ or pseudoalignment with kallisto. ^19^

The config.yaml controls parameters such as thread usage for individual steps, tool-specific parameters, and parameters relating specifically to the A2TEA.Workflow. Two other very important choices that have to be considered are whether or not automatic filtering for the longest representative isoforms of the peptide FASTA files should be performed and whether gene or transcript level quantification is wanted. Choosing automatic isoform filtering will create a subset peptide FASTA file with only the longest isoform per gene; the header will be shortened to just the gene name identifier. This option must be used in conjunction with gene level quantification since otherwise matching both types of data is not possible.

The notion behind the hypotheses.tsv table is outlined in the Implementation section due to its central importance to the expansion calculation. Here, we briefly want to present some of the available choices the user can consider. Besides defining sets of species that should be analyzed for expansion compared to other sets of species, the user is able to specify the required numerical differences between the two and which OGs to disregard immediately. For instance, “Nmin_expanded_in” takes as input an integer value that defines the minimum number of the investigated species that need to fulfill the expansion criteria in order for the gene family to be called “expanded”. These criteria are for instance “min_expansion_factor” and “min_expansion_difference” with which either the minimum factor of expansion or the minimum number of additional genes compared to the non-expanded set of species can be defined. To complement these broad cutoffs, the workflow also integrates a hypothesis-specific CAFE analysis, with which changes in gene family size are analyzed in a way that accounts for phylogenetic history and provides a statistical foundation for evolutionary inferences. ^20^

After all choices have been made, the workflow can be started with a single Snakemake command. A2TEA.Workflow will then perform all previously listed steps and merge results into the final output file described in the Implementation section ( Figure 2). The user is then able to investigate the integrated and condensed results.

We offer several ways of starting an A2TEA.WebApp instance for downstream investigation of the data: 1) installation with R devtools from our GitHub repository, 2) a docker container with the latest release installed, and lastly, 3) a demo instance hosted on shinyapps.io ( https://tgstoecker.shinyapps.io/A2TEA-WebApp/). As the A2TEA.WebApp is an interactive tool with an explorative focus and no strict work order, we illustrate its core operative features in the dedicated Use cases section of this manuscript.

## Use cases

In this section, we will illustrate the functionality of the A2TEA.WebApp, using the A2TEA.Workflow results of a three-species analysis of *Hordeum vulgare* (barley), *Zea mays* (maize), and *Oryza sativa japonica* (rice) that investigates adaptive processes in barley to drought stress. Details on the files used as well as their respective publication and SRA accession numbers are listed in detail in both GitHub repositories and the Source data section. ^48^^,^^49^^,^^50^

We integrated this dataset into the workflow and the web application to illustrate the software’s setup and to allow for a quick exploration of the tools’ functionalities. After cloning the A2TEA.Workflow repository, an additional script can be run (get_test_data.sh) that quickly sets up the experiment by downloading the required input files. Peptide FASTA files are reduced to 2000 proteins; the transcriptomic data is subsampled to 2M reads to allow for a quicker runtime. The functional annotations are precomputed by AHRD. The differential expression analysis is set to be performed on the gene level and two comparisons are performed as defined in the hypotheses.tsv table. These are “Expanded in barley compared to rice and maize” and “Expanded in barley compared to maize”. For both, expansion is defined as "number of genes species A ≥ 2 × number of genes of species B”.

The final output produced by the workflow is also integrated into the current release of the A2TEA.WebApp and can be loaded via clicking the “Try a demo A2TEA.RData file” at the top of the interface.

### Initial inspection of integrated data

The general analysis tab is the default view inside the A2TEA.WebApp. Once input is loaded, reactive information boxes display the number of species, the number of expanded OGs, and the number of DEGs for the currently selected hypothesis. Changing the hypothesis ( Figure 3B) e.g., to the second hypothesis in our test set (“Expanded in barley compared to maize”), changes the statistics and all other sets/plots to reflect only the species considered in the hypothesis. Two tables display gene-level differential expression results and functional annotation information (human readable descriptions and GO terms), which allow, for example, the exploration of genes related to a particular function. Also displayed are an inferred phylogenetic tree of the species in the hypothesis subset and an intersection plot (Venn/UpSet) which displays the number of conserved (OG with ≥1 gene from every species), overlapping, or species unique OGs and singleton genes. Importantly, a table describing the details of the currently displayed hypothesis is also displayed. All of this facilitates a broad overview of the data and allows the user to spot errors such as faulty hypothesis definitions or cutoffs that are too strict.

### Exploring expansion events with annotated phylogenetic trees

The main feature of the TEA (trait-specific evolutionary adaptation) tab is a comprehensive toolkit for the visualization of maximum likelihood phylogenies of expanded OGs and associated information such as the log2(fold change) of the displayed genes and an MSA of the respective protein sequences ( Figure 3). The MSA can be added as a geometric layer to the tree plots or displayed separately with additional options such as a conservation bar ( Figure 3A). To make an informed decision of which OGs are most worthwhile to investigate closer, a table showcasing the total and significantly differentially expressed genes per OG is also provided. With this, the user is enabled to apply several filters, for example, to select all expanded OGs that possess at least 1 DEG and more than 4 genes from *Hordeum vulgare.* The last table on the tab provides insight into the reciprocal BLAST/DIAMOND hits for the currently chosen OG and the additional most similar OGs. Notably, this table also provides the identifiers given to the proteins by Orthofinder, ^21^ making it easy to relate insight gained in the web application back to other outputs created by Orthofinder in the A2TEA.Workflow, such as the list of putative xenologs.

### Comparing sets of orthologous groups

**Figure 4.**
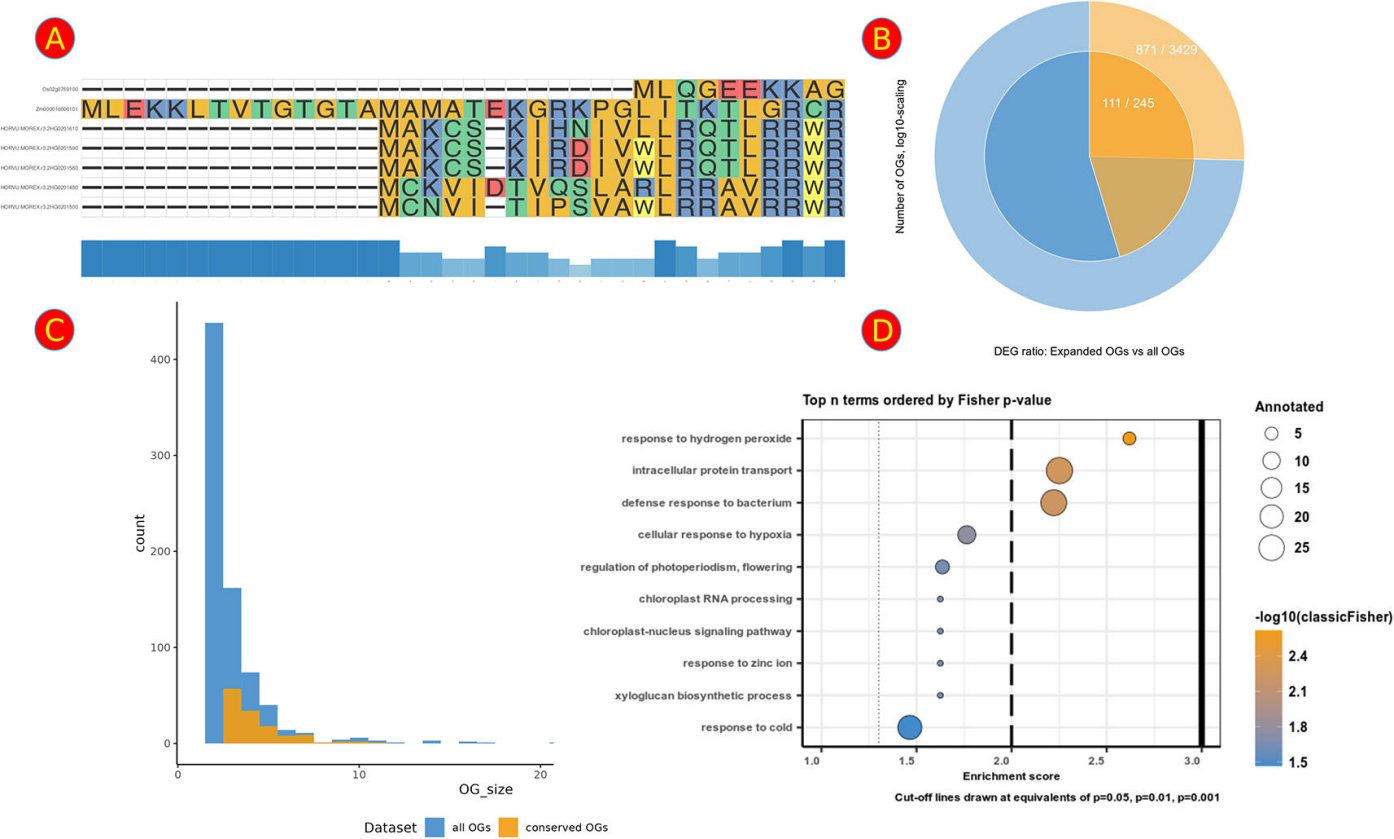
Overview of several analysis plots featured in the A2TEA.WebApp. (A) Multiple sequence alignment of an expanded orthologous group (OG) + additional most similar singletons or OGs. Bars at the bottom represent the degree of conservation. (B) Visual representation of a hypergeometric test for over-representation - colors/layers represent sets in the urn model. The outer ring shows the background set (light blue; “complete background”) and the subset in the background set (light orange; “success in background”). The inner ring displays the set of interest (blue; “sample size”) and the subset in the set of interest (orange; “success in sample”). (C) Barplot of the total number of OGs per group size (number of genes) between all OGs (blue) and only those OGs that are conserved among the analyzed species (orange). (D) Dotplot of the top over-represented biological processes in the subset of OGs of interest compared to the background; dot size indicates the number of OGs with the respective GO term; color displays negative log [base 10] of the p-values from the enrichment test.

To describe adaptive processes at a larger scale, we also integrated functionality to visualize distributions of user-defined OG sets and test for their over-representation; e.g., “What is the frequency of OGs that show expansion and at least 1 DEG in Hordeum vulgare in all conserved OGs?” and “Is this set over-represented within the background distribution of conserved OGs with at least 1 DEG from any species?”. We took care to make answering such questions very accessible by providing the user with text-based choices of which sets to plot or compare. Currently integrated are an enrichment analysis suite allowing for Fisher-Tests and a corresponding circular set plot ( Figure 4B) that visualizes the chosen sets. Also provided is a tool for comparing the size distributions of the OGs ( Figure 4C) with which group size effects can be checked; e.g., “Do we see differences in the number of DEGs in OGs of a certain size range between the set of interest and the background set?”.

### Performing functional enrichment tests

The last analysis tab provides options for performing GO term over-representation analysis based on the topGO R package. ^22^ Functions that occur more often than expected can be identified by setting several parameters that specify the set of OGs the user wants to analyze. With our test data, the user could, for instance, be interested in enriched molecular functions of OGs that are expanded in Hordeum vulgare and also possess at least 2 DEGs of Hordeum vulgare. Once computed, a table is displayed that shows the top significantly enriched GO terms and also contains dynamically created links for these to AmiGO2. ^11^ A second table contains information on the corresponding OGs and genes so that the user can follow up on a particular enriched GO term and inspect the underlying data. We also provide two visualizations that summarize the results. The first is a GO enrichment dotplot ( Figure 4D) straightforwardly showcasing the overall results, and the second is a GO subgraph of selected top N enriched GO terms. With the latter, we provide the user with an insightful way of investigating how the significant GO terms are distributed over the GO graph.

### Export options, bookmarking & ending a session

Tables can be downloaded as .tsv files, and plots are exportable into various formats, such as.pdf, .png, or.svg, allowing the user to easily save and share the observations and results. However, even a relatively small set of species, like the three Poaceae species in our test data, lead to several OGs that are worthwhile to investigate, substantiating the need for the bookmarking system outlined in the Implementation section. It quickly becomes very valuable to bookmark, e.g., all OGs annotated with the top 5 enriched BP GO terms in the OGs expanded in *Hordeum vulgare* if the intention is to return to the analysis later or to generate a list to use with another tool quickly. Relating this to the previous sub-sections, we want to emphasize that bookmarking is integral to using the A2TEA.WebApp and is fully featured on all analysis tabs except the “Set analyses” since here individual genes or OGs are not the focus. To further aid users in the bookmarking process, we also added informative pop-up messages to indicate for instance, that all selected genes/OGs have already been saved. Since the bookmarks can also be used to export a completely functional .RData subset file, only the most relevant information is kept while the processing speed is increased, and all relevant results of the integrative effort are kept. If, for instance, during the analysis, it turned out that hypothesis 2 in our example data (“Expanded in barley compared to maize”) is, in fact, not of interest anymore, subsetting the .RData file to interesting OGs of hypothesis 1 completely removes the unneeded “bloat” of hypothesis 2. Similarly, the user could create 2 .RData files (one for each hypothesis) and run a custom script on each separately, efficiently producing hypothesis-specific results.

## Discussion

Classic transcriptomic studies produce large lists of gene regulatory information for which, traditionally, pathway or GO term analyses are used to discover the overall molecular trends caused by the experimental treatments. ^23^ We propose that we can identify novel genes relevant for stress adaptation by comparing same-stress experiments of several plant species with different levels of stress adaptation in combination with evolutionary footprints in the form of protein family expansion. As illustrated in the Methods & Use Cases sections, our novel software tool A2TEA facilitates the identification of genes associated with the evolution of a trait in a species or a group of related species. Based on the rediscovery of known genes related to the trait, we believe that also novel genes discovered through A2TEA are related to the trait, but experimental verification is in progress, see below. As an example, Figure 3 presents a possible subfunctionalization of gene duplicates in *Eutrema salsugineum*, discovered from data of drought tolerance among four Brassicaceae species (details see Underlying Data). The *A. thaliana* homolog is involved in drought stress response. ^24^

Several approaches have been employed to identify potential candidate genes that could provide a genetic basis for more resilient crops. This includes forward genetics approaches such as identifying causative genes for advantageous mutant phenotypes, ^25^ finding common regulators for several stresses via traditional transcriptomics, ^26^ usage of Quantitative Trait Locus (QTL) mapping and Genome-wide association studies (GWAS) incl. potential integration with expression data, ^27^ the combination of expression data with functional information and clustering methods ^28^^,^^29^ and also machine learning based approaches that employ transcriptomic or phenomic data as the basis of their candidate gene predictions. ^30^^,^^31^

The underlying methods are manifold and include approaches such as Bulked-Segregant analysis, ^32^ k-means clustering, ^33^ WGCNA, ^34^ co-expression networks ^35^ and set analyses of DEGs often in combination with pathway or GO term enrichment analyses. ^23^ While most studies share the approach of reducing a list of regulated genes via secondary criteria, to our knowledge, A2TEA is the first openly available tool that specifically combines stress-specific expression data from several species with gene family expansion to unravel candidate genes for stress adaptation in stress-tolerant species.

With A2TEA, we present software that simplifies the complex bioinformatics workflow for the user and provides an interactive web interface for analysis of the results. By using Snakemake as a bioinformatic workflow manager, we remove the need for step-by-step handling of raw data (including software setup and dependencies necessary for computations) and ensure FAIR (findable, accessible, interoperable, and reusable) computational analysis standards. ^36^ The downstream analysis and visualization framework makes the navigation of the resulting large sets of tabular data faster, more intuitive, and more practicable for scientists without programming skills. ^12^^,^^23^ It offers a variety of summary statistics on the levels of gene family expansion, differential expression, and functional enrichment to ensure quality control. The Shiny framework provides interactivity regarding the visualization and the analysis of the results, and this interactivity highly facilitates the exploration of scientific questions.

Based on user experiences with the web application, we have included analyses and visualizations to allow detecting problems in the bioinformatic predictions, e.g., of orthologous groups (OGs). In order to spot potential misassignments of Orthofinder, close homologs to members of an OG are detected by similarity search and displayed with phylogenetic trees and multiple sequence alignments. A typical case is the non-inclusion of a singleton gene of a species due to a significant portion of protein sequence missing in the annotation, caused e.g., by gaps or sequencing errors in the genome sequence or errors in gene prediction. Similarly, false expansions based on a putative paralog that has only very limited alignment overlap with other members of the OG can be detected. These could be actual duplicates but degenerated through pseudogenization or partial duplication, e.g., the action of transposable elements.

We designed A2TEA with extendability in mind. Both the Snakemake-based A2TEA.Workflow and the A2TEA Shiny App can be easily expanded in a modular fashion to integrate novel features. We are currently testing several additional visualization and testing options. This includes the option for positive selection tests concerning a particular OG, e.g., by calculating the ratio of non-synonymous amino-acid substitutions over synonymous amino-acid substitutions (dN/dS)), distribution comparisons between random and actual DEG-containing OGs, and visualizations for the analysis of general gene/transcript regulation trends. The GO term enrichment functionality is aimed at discovering general trends in the adaptation to the particular stress under investigation. At the moment, the implemented enrichment tests provide options for single over-representation analysis as implemented in the R topGO package. ^22^ It will be interesting to evaluate and potentially implement further options for functional enrichment analysis in A2TEA, such as modern ensembl approaches ^37^ or simplification strategies that aid in summarization. ^38^ Lastly, we intend to implement the option to download a comprehensive RMarkdown/Quarto report summarizing plots and statistics for all bookmarked genes and OGs. This has been demonstrated to be a significant step forward in guaranteeing the portability of results once an interactive session is concluded. ^12^

While our research focus is on crops, from a software perspective, A2TEA is entirely independent of the underlying species and can be used with any set of species. This opens the question of how feasible applying the A2TEA methodology to species from other kingdoms might be. Our motivation for developing A2TEA is primarily rooted in the notion that genome duplication played a major role in the evolutionary past of plants. ^4^ Plant comparative genomics research has shown that gene families are mostly conserved across great evolutionary timescales, comprising even the diversification of all angiosperms and nonflowering plants. ^39^^,^^40^ Fascinatingly, this conservation of gene families is combined with lineage-specific fluctuations in gene family size, which are frequent among taxa. ^4^^,^^39^^–^^42^ This suggests that since comparatively few novel gene families arose, much of the great diversity and phenotypic variation seen in land plants may have arisen primarily due to duplication and adaptive specialization of already existing genes. ^40^

While whole genome duplication events are expected and reported less frequently in the animal kingdom and thus gene duplication as a driver of protein family expansion does not play as prominent a role in animals as in plants, ^43^^,^^44^ protein family expansion is still an important driver of adaptation. ^45^ We expect that A2TEA will be useful in non-plant species, even if protein family expansion only represents a small portion of adaptive changes, with other sources of variation, like alternative splicing playing a potentially more important role. ^46^

Currently, we are investigating several publicly available genomic and transcriptomic datasets from various groups of plant species with A2TEA. While we expect to detect candidate genes relevant to adaptation to the stress being investigated, this assumption is based on the rediscovery of known genes. One important follow-up step is thus to experimentally verify the impact of selected candidate genes in vivo. To this end, we perform stress experiments in plants bearing knockout mutations in candidate genes predicted by A2TEA, using sequence-indexed mutant collections such as BonnMu. ^47^ This will allow us to assess the phenotypic impact of these mutations and, thus, the role of these genes in the tolerance of the stress. While testing all candidates will not be feasible, the rate of genes relevant to the trait under investigation among tested candidates will represent an estimate of the prediction performance.

## Conclusions

With the availability of multiple genome sequence and RNA-seq data sets, it is now possible to combine comparative evolutionary analyses, in our case protein family expansion, with differential expression to predict genes involved in adaptive traits. However, running the required bioinformatics analyses and data integration tasks as well as summarizing and visualizing the results, remains challenging. A2TEA only requires standard data files as input, follows best practice software standards for both reproducibility and portability, and provides a user-friendly web application for interactive exploration and selection of the most promising candidate genes. We show that genes known to be involved in stress tolerance can be detected in datasets of stress-tolerant and stress-sensitive plants, but we expect A2TEA to be useful in a broader scope when analyzing protein families and their expression in multiple genomes as the parameters for selecting interesting families are very flexible. A2TEA follows a positive trend in modern research software development that provides easy installation and execution through the use of container and workflow technologies as well as interactive visualization and exploration tools for the generated results. Combined, this facilitates better reproducibility, communication, and shareability of comprehensive analyses.

## Data availability

### Source data

**Poaceae test data**:

Transcriptomics:

*Hordeum vulgare*: SRR6782243, SRR6782247, SRR6782257, SRR6782249, SRR6782250, SRR6782254

*Zea mays*: SRR2043219, SRR2043217, SRR2043190, SRR2043220, SRR2043226, SRR2043227

*Oryza sativa japonica*: SRR5134063, SRR5134064, SRR5134065, SRR5134066

These correspond to the following studies relating on drought stress:

*Hordeum vulgare*: https://doi.org/10.1186/s12864-019-5634-0

*Zea mays*: https://doi.org/10.1104/pp.16.01045

*Oryza sativa japonica*: https://doi.org/10.3389/fpls.2017.00580

Assemblies & annotations hosted on EnsemblPlants:

*Hordeum vulgare*: cDNA FASTA, GTF, Peptide FASTA

*Zea mays*: Genome FASTA, GTF, Peptide FASTA

*Oryza sativa japonica*: cDNA FASTA, GTF, Peptide FASTA

An archived version of the complete grasses test data (reduced as used in the examples) is deposited here:

https://zenodo.org/record/7089022 ^48^

**Data used in the Brassicaceae example:**

Transcriptomics:

*Eutrema salsugineum*: SRR7624684, SRR7624685, SRR7624692, SRR7624687, SRR7624721, SRR7624722

*Arabidopsis lyrata*: SRR7624680, SRR7624702, SRR7624703, SRR7624732, SRR7624733, SRR7624742

*Arabidopsis thaliana*: SRR7624694, SRR7624696, SRR7624697, SRR7624710, SRR7624714, SRR7624723

*Brassica napus*: SRR12429701, SRR12429702, SRR12429703, SRR12429698, SRR12429699, SRR12429700

These correspond to the following studies on drought stress response: *Eutrema salsugineum, Arabidopsis lyrata, Arabidopsis thaliana*: https://doi.org/10.1111/nph.15841

*Brassica napus*: PRJNA656507

Assemblies & annotations hosted on EnsemblPlants:

*Eutrema salsugineum*: Genome FASTA, GTF, Peptide FASTA

*Arabidopsis lyrata*; Genome FASTA, GTF, Peptide FASTA

*Arabidopsis thaliana*: Genome FASTA, GTF, Peptide FASTA

*Brassica napus*: Genome FASTA, GTF, Peptide FASTA

### Underlying data

The results generated by the A2TEA.Workflow which are also used for demonstrating the A2TEA.WebApp’s functionality presented in this work are available at https://zenodo.org/record/7089608 ^49^ and https://zenodo.org/record/7089606. ^50^

Data are available under the terms of the Creative Commons Attribution 4.0 International license (CC-BY 4.0).

## Software availability

Both the A2TEA.Workflow and the A2TEA.WebApp are available as MIT licensed open source softwares.

• Software available from: https://tgstoecker.github.io/A2TEA.WebApp
• Source code available from: https://github.com/tgstoecker/A2TEA.Workflow, https://github.com/tgstoecker/A2TEA.WebApp
• Archived source code at time of publication: https://zenodo.org/record/7086290, ^51^ https://zenodo.org/record/7086282 ^52^
• License: MIT
