# Supplementary material for: A2TEA: Identifying trait-specific evolutionary adaptations
Source: F1000Res. 2023 Apr 12;11:1137. Originally published 2022 Oct 5. [Version 2] doi: 10.12688/f1000research.126463.2 (PMC10186066; doi:10.12688/f1000research.126463.2)
Supplement: Peer review history

# Peer review: Reviewer response for version 2

**DOI:** 10.5256/f1000research.146089.r169362

**Published:** 2023-05-15

**Authors:** Rahul Siddharthan

**Affiliations:** 1 Computational Biology, The Institute of Mathematical Sciences, Chennai, Tamil Nadu, India

I am satisfied by the changes made by the authors and have no further comment.

Are the conclusions about the tool and its performance adequately supported by the findings presented in the article?

Yes

Is the rationale for developing the new software tool clearly explained?

Yes

Is the description of the software tool technically sound?

Yes

Are sufficient details of the code, methods and analysis (if applicable) provided to allow replication of the software development and its use by others?

Yes

Is sufficient information provided to allow interpretation of the expected output datasets and any results generated using the tool?

Yes

Reviewer Expertise:

Computational biology: regulatory genomics, chromatin, algorithms

I confirm that I have read this submission and believe that I have an appropriate level of expertise to confirm that it is of an acceptable scientific standard.

# Peer review: Reviewer response for version 2

**DOI:** 10.5256/f1000research.146089.r169361

**Published:** 2023-05-03

**Authors:** Manuel Aranda, Octavio Salazar Moya

**Affiliations:** 1 Biological and Environmental Sciences and Engineering Division (BESE), Red Sea Research Center (RSRC), King Abdullah University of Science and Technology (KAUST), Thuwal, Saudi Arabia

The authors addressed most of our comments adequately and we have only a few comments left.

 Specific comments:

 1 . Introduction: “These approaches thus have clear limitations when used in isolation.” We suggest changing it to: These approaches, thus, have clear limitations when used in isolation.

 2. Related to our previous comment:

 “GO term analyses tab: At GO term analyses, changing the algorithm does not change the column name at GO term set choices. It always says classicFisher. Same in enrichment plots. Also, in the enrichment plots, it might be better to plot the cut-off lines after plotting the terms, as sometimes the terms are small due to a low number of annotated genes belonging to them and might be covered by the line. Changing the x-axis label for the bar plot to the number of significant orthogroups might be better.”

 The authors answered:

 The choice of the topGO algorithm concerns how the GO graph is analyzed (independence vs. consideration parent terms, etc.) and is a separate option from the downstream test statistic (https://bioconductor.org/packages/release/bioc/vignettes/topGO/inst/doc/topGO.pdf). In the current release, we only allow classicFisher as the test statistic. As such, the column does not need to change in our opinion. However, we agree that information of the used algorithm should be kept. We have now implemented that the name of the algorithm is part of the output tables that the user is able to download.

 New comment:

 The authors mention that only classicFisher is allowed as the test statistic. However, under GO term set choices, there is a drop down menu allowing the user to specify the Algorithm as classic, elim, weight, weight01, and parentchild. Changing this option leads to different results at the Go term set choices window (Also Go should be changed to GO), suggesting that the current release of A2TEA does support the use of other algorithms other than classicFisher. Moreover, the p-values do change. Which would then come back to our previous comment of having the column name change according to the algorithm used at the Go term set choices output.

Are the conclusions about the tool and its performance adequately supported by the findings presented in the article?

Yes

Is the rationale for developing the new software tool clearly explained?

Yes

Is the description of the software tool technically sound?

Yes

Are sufficient details of the code, methods and analysis (if applicable) provided to allow replication of the software development and its use by others?

Yes

Is sufficient information provided to allow interpretation of the expected output datasets and any results generated using the tool?

Yes

Reviewer Expertise:

Genomics

We confirm that we have read this submission and believe that we have an appropriate level of expertise to confirm that it is of an acceptable scientific standard.

# Peer review: Reviewer response for version 1

**DOI:** 10.5256/f1000research.138877.r156575

**Published:** 2023-02-01

**Authors:** Rahul Siddharthan

**Affiliations:** 1 Computational Biology, The Institute of Mathematical Sciences, Chennai, Tamil Nadu, India

The software tool A2TEA is well described and motivated. The idea of identifying genes involve in adaptive traits, such as stress-tolerance, using inter-species comparisons, is sound. A2TEA integrates the bioinformatics (RNA-seq, orthologous group computation, functional information inference, etc.

 I do not work in this field so have not tested out the tool. The webapp looks well laid out and user-friendly but I did not try it on actual data.

 Several boxes in the flowchart in figure 2 have garbled text (text replaced with boxes).  I also suggest highlighting a couple of possible paths through this figure in an actual workflow (eg, a single workflow would not use both kallisto and STAR?).

 Minor comment: one block of text, starting with "The A2TEA.WebApp is written in the R programming language..." (two paragraphs) is repeated (as a single paragraph immediately after).

Are the conclusions about the tool and its performance adequately supported by the findings presented in the article?

Yes

Is the rationale for developing the new software tool clearly explained?

Yes

Is the description of the software tool technically sound?

Yes

Are sufficient details of the code, methods and analysis (if applicable) provided to allow replication of the software development and its use by others?

Yes

Is sufficient information provided to allow interpretation of the expected output datasets and any results generated using the tool?

Yes

Reviewer Expertise:

Computational biology: regulatory genomics, chromatin, algorithms

I confirm that I have read this submission and believe that I have an appropriate level of expertise to confirm that it is of an acceptable scientific standard, however I have significant reservations, as outlined above.

# Response

**Published:** 2023-03-24

**Authors:** Tyll Stöcker

We thank all reviewers for providing valuable input to the article that helped us to improve it further. We have tried to address their comments and made efforts to incorporate their insightful criticisms and suggestions. Similar suggestions and criticisms have been grouped together in order to address them concisely.

- **Several boxes in the flowchart in figure 2 have garbled text (text replaced with boxes). I also suggest highlighting a couple of possible paths through this figure in an actual workflow (eg, a single workflow would not use both kallisto and STAR?).**
  We have made sure that the figure is now without the noted garbled text. Regarding the paths through the diagram: The reviewer is correct that in a normal differential expression analysis one would not choose to process some of the samples with one tool and the rest with another. However, in our field of plant/crop research, we have encountered many cases of early assembly and annotation versions, with many cases of only a transcriptome or a genome assembly being available. Due to this, the workflow does support running one species through a pseudoalignment and another through classic alignment-based quantification to provide more flexibility when combining data from different sources. In our Workflow README, we point out that for runtime and resource purposes, we recommend kallisto/pseudoalignment, and it should always be preferred if possible.
- **Minor comment: one block of text, starting with "The A2TEA.WebApp is written in the R programming language..." (two paragraphs) is repeated (as a single paragraph immediately after).**
  We thank the reviewer very much for pointing out this mistake that occurred during our editing process.

# Peer review: Reviewer response for version 1

**DOI:** 10.5256/f1000research.138877.r154237

**Published:** 2022-11-25

**Authors:** Manuel Aranda, Octavio Salazar Moya

**Affiliations:** 1 Biological and Environmental Sciences and Engineering Division (BESE), Red Sea Research Center (RSRC), King Abdullah University of Science and Technology (KAUST), Thuwal, Saudi Arabia

General comments:

 The manuscript submitted by Stöcker et *et al*. describes an integrated pipeline for the identification of genes potentially involved in adaptation by analyzing gene duplications and gene family expansions. The pipeline combines RNA-Seq expression analyses via DESeq2, identification and phylogenetic analysis of orthologous groups with OrthoFinder, statistical analyses of gene family changes taking into account phylogeny inference with Cafe5, and gene ontology enrichment analyses with TopGO. The results of the pipeline can be visualized using the R shiny package, which allows for easy exploration of the results even for experimentalists with little bioinformatic background. A2TEA is a simple yet useful tool for the visualization and integration of multiple analyses and datasets. A few things need to be revised in the manuscript, such as a corrupted Figure 2, repeated paragraphs, and the addition of missing references. The implementation of a “Download All Results” option in the general tab of the shiny app would be helpful. Allowing the pipeline to run without the addition of RNA-Seq could be quite useful to allow analyses of species without available RNA-Seq data from the same condition. Additionally, it would be appropriate to put an emphasis on the broader use of the app instead of focusing on plant stress, as it would make the app more appealing to a broader audience.

Specific comments

- Abstract: “It functions as a one-stop processing pipeline, integrating protein family, phylogeny, expression, and protein function analysis”. Change analysis to analyses.
- Abstract: “The pipeline is accompanied by an R Shiny web application that”. Jump in line, fix editing.
- Introduction: “Most of the duplicates are lost or silenced….”. Maybe change it to something like: Most of the gene duplicates are lost or silenced, but retained duplicates may hint at some evolutionary advantage, which may be targets of adaptation.
- Introduction: The first paragraph of the introduction needs some work. It is unclear if the authors are explaining the different approaches that can be used to identify genes related to adaptation or if they are referring to their pipeline, as they use “we” and “us”. It would be appropriate to first describe the different approaches and their benefits and then the need to combine them. The second and third paragraphs already properly address what the pipeline is doing.
- Introduction: Change & for and at: “working in tandem to automate and ease all bioinformatics & analysis tasks involved.”
- Introduction: Change “gene families in form of” to gene families in the form of…
- Figure 1 (and the whole article). While the tool was designed for the identification of genes for crop improvement, it is not limited to that application. As the name of the application indicates, it is designed for trait-specific evolutionary adaptations. It might be a good idea to generalize the possible applications of this tool rather than focus only on plant stress-specific responses. The authors can specify that an example of its use is in crop improvement while making sure to maintain a general tone for the use of A2TEA for a broader audience.
- Cite DIAMOND, DESeq2, BLAST.
- Cite Cafe in the main text (only found in the reference list).
- This paragraph is repeated: “The A2TEA.WebApp is written in the R programming language and uses the Shiny framework to facilitate interactivity with the data. It expects the user to upload a .RData file created by the A2TEA.Workflow. The web application comes with a test dataset that can be loaded with a single click so interested users can try out its functionality before having to finish an A2TEA.Workflow run.”
- Figure 2 is full of typos like orthoander instead of orthofinder and many of the boxes don’t have text but empty squares, probably an error while formatting.
- Figure 3. Italicize Eutrema salsugineum.
- Figure 4. Increase the resolution or the font size, as words in panels B, C, and D are blurry. 
- Comments on the A2TEA Shiny App.
  - General tab: Differential expression and Functional annotation require a “Download Full Results” button and not only a download current page.
  - GO term analyses tab: At GO term analyses, changing the algorithm does not change the column name at GO term set choices. It always says classicFisher. Same in enrichment plots. Also, in the enrichment plots, it might be better to plot the cut-off lines after plotting the terms, as sometimes the terms are small due to a low number of annotated genes belonging to them and might be covered by the line. Changing the x-axis label for the bar plot to the number of significant orthogroups might be better.
- Discussion: “We propose that we can identify novel genes relevant for stress adaptation by comparing same-stress experiments of several plant species with different levels of stress adaptation in combination with evolutionary footprints in the form of protein family expansion”. Again, it would be better to keep the usability of this tool as general as possible, not only focusing on plat stress, as it could be used for any species and conditions. Also, using the word condition or treatment instead of stress would be better.
- Additional comments. It could be beneficial if the App could also be run without RNA-Seq data for the development of a general hypothesis when comparing species for which RNA-Seq data under the same conditions is not available.

Are the conclusions about the tool and its performance adequately supported by the findings presented in the article?

Yes

Is the rationale for developing the new software tool clearly explained?

Yes

Is the description of the software tool technically sound?

Yes

Are sufficient details of the code, methods and analysis (if applicable) provided to allow replication of the software development and its use by others?

Yes

Is sufficient information provided to allow interpretation of the expected output datasets and any results generated using the tool?

Yes

Reviewer Expertise:

Genomics

We confirm that we have read this submission and believe that we have an appropriate level of expertise to confirm that it is of an acceptable scientific standard, however we have significant reservations, as outlined above.

# Response

**Published:** 2023-03-24

**Authors:** Tyll Stöcker

We thank all reviewers for providing valuable input to the article that helped us to improve it further. We have tried to address their comments and made efforts to incorporate their insightful criticisms and suggestions. Similar suggestions and criticisms have been grouped together in order to address them concisely.

- **Abstract: “It functions as a one-stop processing pipeline, integrating protein family, phylogeny, expression, and protein function analysis”. Change analysis to analyses.**
- **Abstract: “The pipeline is accompanied by an R Shiny web application that”. Jump in line, fix editing.**
- **Introduction: “Most of the duplicates are lost or silenced….”. Maybe change it to something like: Most of the gene duplicates are lost or silenced, but retained duplicates may hint at some evolutionary advantage, which may be targets of adaptation.**
- **Introduction: Change & for and at: “working in tandem to automate and ease all bioinformatics & analysis tasks involved.”**
- **Introduction: Change “gene families in form of” to gene families in the form of…**
  We thank the reviewer very much for taking the time and point out several minor formatting and phrasing errors, all of which we have corrected.
- **Introduction: The first paragraph of the introduction needs some work. It is unclear if the authors are explaining the different approaches that can be used to identify genes related to adaptation or if they are referring to their pipeline, as they use “we” and “us”. It would be appropriate to first describe the different approaches and their benefits and then the need to combine them. The second and third paragraphs already properly address what the pipeline is doing.**
  On re-reading the first paragraph of the introduction with the reviewer's comments in mind, we strongly agreed that a more logical separation was necessary. We have restructured and rewritten parts of it to more clearly separate the prior approaches and then – in a second step – the need to combine them as we do with our software.
- **Cite DIAMOND, DESeq2, BLAST. **
- **Cite Cafe in the main text (only found in the reference list).**
  We thank the reviewer for pointing out important missing citations in our text and have added these as well as additional ones. We have repositioned our citation of CAFE5 immediately next to the software’s name in the text (prior it was at the end of the sentence).
- **This paragraph is repeated: “The A2TEA.WebApp is written in the R programming language and uses the Shiny framework to facilitate interactivity with the data. It expects the user to upload a .RData file created by the A2TEA.Workflow. The web application comes with a test dataset that can be loaded with a single click so interested users can try out its functionality before having to finish an A2TEA.Workflow run.”**
  We thank the reviewer very much for pointing out this mistake that occurred during our editing process.
- **Figure 2 is full of typos like orthoander instead of orthofinder and many of the boxes don’t have text but empty squares, probably an error while formatting.**
  Unsure of the cause, we have uploaded the figure in a different format which seems to have solved the garbled text.
- **Figure 3. Italicize Eutrema salsugineum.**
  We italicized this species in the figure text and took care to check the manuscript for similar formatting flaws.
- **Figure 4. Increase the resolution or the font size, as words in panels B, C, and D are blurry.**
  We re-exported the image in higher resolution to increase the overall legibility.
- **Comments on the A2TEA Shiny App.**
**General tab: Differential expression and Functional annotation require a “Download Full Results” button and not only a download current page.**
  The reviewer was correct in pointing out the need for this feature. For all tables, we have now implemented the possibility of downloading all results and not just the displayed page via the suggested buttons. If the user did not perform any filtering, the complete table is downloaded; if the user performs filter operations (e.g. | log2FC | > 1) the complete, filtered table is downloaded.
**GO term analyses tab: At GO term analyses, changing the algorithm does not change the column name at GO term set choices. It always says classicFisher. Same in enrichment plots. Also, in the enrichment plots, it might be better to plot the cut-off lines after plotting the terms, as sometimes the terms are small due to a low number of annotated genes belonging to them and might be covered by the line. Changing the x-axis label for the bar plot to the number of significant orthogroups might be better.**
  The choice of the topGO algorithm concerns how the GO graph is analyzed (independence vs. consideration parent terms, etc.) and is a separate option from the downstream test statistic ( https://bioconductor.org/packages/release/bioc/vignettes/topGO/inst/doc/topGO.pdf). In the current release, we only allow classicFisher as the test statistic. As such, the column does not need to change in our opinion. However, we agree that information of the used algorithm should be kept. We have now implemented that the name of the algorithm is part of the output tables that the user is able to download.
  We have changed the order of the plotting in the enrichment plot – now, the lines plot behind the terms, which ensures clearer visualizations.
  We have also adapted the reviewer's suggestion for a better x-axis label in the enrichment bar plot.
- **Figure 1 (and the whole article). While the tool was designed for the identification of genes for crop improvement, it is not limited to that application. As the name of the application indicates, it is designed for trait-specific evolutionary adaptations. It might be a good idea to generalize the possible applications of this tool rather than focus only on plant stress-specific responses. The authors can specify that an example of its use is in crop improvement while making sure to maintain a general tone for the use of A2TEA for a broader audience.**
- **Discussion: “We propose that we can identify novel genes relevant for stress adaptation by comparing same-stress experiments of several plant species with different levels of stress adaptation in combination with evolutionary footprints in the form of protein family expansion”. Again, it would be better to keep the usability of this tool as general as possible, not only focusing on plant stress, as it could be used for any species and conditions. Also, using the word condition or treatment instead of stress would be better.**
  We agree with the reviewer that the tone and focus of our manuscript can be somewhat loosened to emphasize the general technical feasibility of applying our approach to research outside of the plant kingdom. We have 1. altered parts of the manuscript to incorporate the words treatment/condition instead of the word stress (to underline broader context applicability) and 2. added an additional sentence to the end of the first paragraph emphasizing the general technical applicability in other organisms than plants.
  However, as we point out in two paragraphs near the end of the discussion, our particular approach stems from the support of previous research that genome duplication was a decisive factor in the evolutionary history of plants and much of the phenotypic variation in land plants may have arisen primarily due to duplication and adaptive specialization of already existing genes. Since our method specifically focuses on the analysis of protein family expansion events, it might be much less applicable in other groups of species where other evolutionary processes, such as alternative splicing or pathway/network rewiring, could play a proportionally much more important role. These considerations made us specifically target the "Plant Computational and Quantitative Genomics" collection because the feasibility of our software for research in other kingdoms is unclear.
- **It could be beneficial if the App could also be run without RNA-Seq data for the development of a general hypothesis when comparing species for which RNA-Seq data under the same conditions is not available.**
  We agree with the reviewer's notion of not requiring RNA-seq data for all species in a workflow run. The latest release of the A2TEA.Workflow and WebApp now allow for this. The README of the Workflow explains that if the user does not possess transcriptomic data for a species, they can leave the genomic/cDNA fasta and annotation positions in the species.tsv file empty. We updated the WebApp to handle such cases of missing data. Therefore our whole pipeline has become considerably more flexible.
